# Supplementary material for: Relationship between the surface and internal deformation features of toppling in anti-dip rock slopes
Source: PLoS One. 2024 Nov 7;19(11):e0312687. doi: 10.1371/journal.pone.0312687 (PMC11542880; doi:10.1371/journal.pone.0312687)
Supplement: Attachment — Submitted filename: Comments.docx [file pone.0312687.s010.docx]

**Overall Assessment:**

Reject

**General Overview:**

The manuscript aims at analyzing the failure process of the toppling failure within the anti-dip rock slope, and establishing the relationship between surface behavior and internal fracturing characteristics. The author has conducted extensive research and achieved some meaningful results. Nevertheless, I found some aspects of this paper that lead me to recommend its rejection:

1. The title needs to be rewritten.
2. When an author employs an abbreviation, the full term should appear at the first instance of the abbreviation.
3. “tipping” is suggested to be “toppling”, please check the whole manuscript
4. Insert a space between the unit and the number.
5. Introduction- In fact, the research on the correlation between macroscopic deformation characteristics and internal fractures of anti-dip slopes has been quite comprehensive. On one hand, it is suggested that the authors include references from the past three years concerning studies on toppling failures. On the other hand, the innovative aspects of this paper need further enhancement to emphasize the originality and significance of this research.
6. The manuscript has too many sections, please consolidate some sections to make the structure of the article more concise. For example, Sections 2.1 to 2.4 can be merged into one section.
7. Line 91 Wrong expression “Table 3Ⅰ-a” should be “Figure 3I-a”
8. Line 98 “Observation points were equidistantly placed on the surface” Where are observation points located? Please mark the locations on the figure.
9. Section 2.3. Why the geometric similarity ratio is set of 1:100? What is the prototype rock type of the similar materials? Does the strength of the similar material meet the requirements of prototype rock? Please provide additional details in the manuscript.
10. Line 130 “the max deviator stress（𝜎1−𝜎3）near the foot of the slope is the largest” . Please supplement the stress monitoring data to support this statement.
11. Lines 133-134 “While the small principal stress drops to zero or even changes to tensile stress, the maximum principal stress hits the highest point near the bottom of the slope.” Please supplement the stress monitoring data to support this statement.
12. Line 146 Spelling mistake “slop” should be “slope”.
13. Line 158. “Figure 8” should be “Fig. 7”. In addition, the expression of the figure should be standardized according to the journal's requirements.
14. Line 183 “No. 4” should be “No. 5”.
15. Lines 234-235 “and tension cracks began to form at a degree of stress of about 1°.” I do not understand this sentence, especially the expression “degree of stress of about 1°”. More explanation needed to be added.
16. The content in Section 3.4 is missing.
17. Line 309, “According to the previous centrifugal test, combined with Huang Runqiu’s deformation system”. Which research? What is Huang Runqiu’s deformation system? Please add related references and statement.
18. Line 318. “it is also necessary to subtract 5 cm” Why is 5 cm? It appears that the author intends to correlate the theoretical derivation results with the model test results. However, there seems to be a lack of scientific basis for this approach.
19. Line 322 “Goodman and Bray proposed that the overturning deformation…”. Please add related references.
20. Line 345 The author did not label Zone B in Fig. 12.
21. Fig. 17. What does the term” deformation intensity” mean? In addition, please add a legend to each contour map in Fig. 17.
22. Please add a scale bar to Fig. 21.
23. Line 457, The Zha Yong toppling deformation is in the III stage of slope dumping deformation but without explanation, please supplement. It is recommended that the authors supplement field photographs of slope deformation and compare them with physical model experiments to validate the accuracy of the conclusions. Currently, the classification of toppling evolution stages appears overly subjective, lacking quantitative criteria for assessment and support from field evidence.
24. Linking InSAR with the division of landslide evolutionary stages is a very interesting idea. However, in previous studies, the formation of large-scale toppling deformations in southwest China has been proven to occur during the valley evolution process. In another words, the toppling period is extremely long. Even if you obtain data for a period using InSAR, it is still very short relative to the existence time of toppling.
25. Language- in additon to the terminology issues, there are still a lot of problems regarding the English usage and the expression. The language at places is imprecise. The problems start from the first sentence of Abstract.
