# Supplementary material for: Relationship between the surface and internal deformation features of toppling in anti-dip rock slopes
Source: PLoS One. 2024 Nov 7;19(11):e0312687. doi: 10.1371/journal.pone.0312687 (PMC11542880; doi:10.1371/journal.pone.0312687)
Supplement: Attachment — Submitted filename: Response to Reviewers.docx [file pone.0312687.s011.docx]

Response to Reviewers

**Journal Requirements:**

1. Please ensure that your manuscript meets PLOS ONE's style requirements, including those for file naming. The PLOS ONE style templates can be found at

Answer: Dear Editor, thank you for the reminder, I have revised the formatting of my thesis manuscript

2. We suggest you thoroughly copyedit your manuscript for language usage, spelling, and grammar. If you do not know anyone who can help you do this, you may wish to consider employing a professional scientific editing service.

Answer: Dear Editor, Thank you for the reminder that I have touched up the manuscript of my thesis.

3. Thank you for stating the following in the Acknowledgments Section of your manuscript: "This study is funded by the National Key R&D Program of China (No. 2021YFC3000401)."Please note that funding information should not appear in the Acknowledgments section or other areas of your manuscript. We will only publish funding information present in the Funding Statement section of the online submission form. Please remove any funding-related text from the manuscript.

Answer: I have removed the acknowledgments.

4. Thank you for stating the following financial disclosure: "This study is funded by the National Key R&D Program of China (No. 2021YFC3000401)."  Please state what role the funders took in the study. If the funders had no role, please state: "The funders had no role in study design, data collection and analysis, decision to publish, or preparation of the manuscript." If this statement is not correct you must amend it as needed. Please include this amended Role of Funder statement in your cover letter; we will change the online submission form on your behalf.

Answer: The funders had no role in study design, data collection and analysis, decision to publish, or preparation of the manuscript.

5. We note that your Data Availability Statement is currently as follows: “All relevant data are within the manuscript and its Supporting Information files. “Please confirm at this time whether or not your submission contains all raw data required to replicate the results of your study. Authors must share the “minimal data set” for their submission.

Answer: We're sure that we provided all relevant data, and put the data in supporting information.

6. PLOS requires an ORCID iD for the corresponding author in Editorial Manager on papers submitted after December 6th, 2016. Please ensure that you have an ORCID iD and that it is validated in Editorial Manager. To do this, go to ‘Update my Information’ (in the upper left-hand corner of the main menu), and click on the Fetch/Validate link next to the ORCID field. This will take you to the ORCID site and allow you to create a new iD or authenticate a pre-existing iD in Editorial Manager. Please see the following video for instructions on linking an ORCID iD to your Editorial Manager account: <https://www.youtube.com/watch?v=_xcclfuvtxQ>

Answer: Dear editor, I have applied for ORCID.

7. Please ensure that you refer to Figures 1, and 3 in your text as, if accepted, production will need this reference to link the reader to the figure.

Answer: We accept.

8. We note that Figures 18, 19, and 21 in your submission contain satellite images which may be copyrighted. All PLOS content is published under the Creative Commons Attribution License (CC BY 4.0), which means that the manuscript, images, and Supporting Information files will be freely available online, and any third party is permitted to access, download, copy, distribute, and use these materials in any way, even commercially, with proper attribution. For these reasons, we cannot publish previously copyrighted maps or satellite images created using proprietary data, such as Google software (Google Maps, Street View, and Earth). For more information, see our copyright guidelines: <http://journals.plos.org/plosone/s/licenses-and-copyright>.

SGS National Map Viewer (https://www.usgs.gov/tools/national-map-viewer)
USGS Earth Resources Observatory and Science (EROS) Center (https://www.usgs.gov/centers/eros)
The Gateway to Astronaut Photography of Earth (https://eol.jsc.nasa.gov/)
Maps at the CIA (https://www.cia.gov/the-world-factbook/maps/)
NASA Earth Observatory (https://earthobservatory.nasa.gov/)
Landsat (https://landsat.visibleearth.nasa.gov/)
Natural Earth (https://www.naturalearthdata.com/)
Answer: I have modified the original Fig. 18 to Fig. 2. Since none of the websites you provided provide a map of the prototype of the landslide studied in this article, this article selects the drone orthophoto of the field survey on the drawing of Figure 2. I deleted Figure 19 from the original manuscript. The original Figure 21 is Figure 20 of the new manuscript, using the C-band Sentinel-1A radar satellite imagery launched by ESA for the wide-area surface deformation detection satellite in the study area, and the archived data is free for all users. Figure 21 in the new manuscript is a photograph taken with a fieldwork camera and a drone

**Additional Editor Comments:**

Reviewer #1:

1. Language and Readability: I would suggest the authors have it checked preferably by a native English-speaking person to avoid any mistakes. Also, the manuscript should be revised for technical terms and typographical errors. Lastly, ensure consistent use of terms.

Answer: Dear reviewer, we have revised the article on the issues you raised and touched up the article.

2. Line 99: Why was the toe of the slope model cut off? What is the basis for this decision?

Answer: The slope prototype is located in the Jinsha River basin, and cutting the foot of the modeled slope simulates the erosion of the foot of the slope prototype due to the Jinsha River scour.

3. Lines 101-104: I did not find information regarding the prototype slope in the manuscript. How were the geometric similarity ratio and the physical-mechanical parameters determined?

Answer: Thanks for your kind reminders. We have added the information of the prototype slope, taking into account the capacity of the centrifuge, the size of the model box and the maximum centrifugal acceleration, the maximum centrifugal acceleration used in the test was finally determined to be 100g.

4. Line 128-140: In the analysis of experimental phenomena, the manuscript describes changes in the maximum and minimum principal stresses at the slope toe. However, this phenomenon is not clearly evidenced in the model experiments. Please provide an explanation for this.

Answer：Thanks for your kind reminders. According to Principles of Engineering Geological Analysis and Engineering Geology: Rock in Engineering Construction, during slope formation, the rock mass around the critical surface undergoes unloading and rebound, causing stress redistribution and stress concentration effects. Under the influence of stress differentiation, a stress concentration zone is generated near the critical face. The maximum principal stress near the foot of the slope increases and the minimum principal stress decreases, so that the difference between the maximum and minimum principal stresses here is the largest, which makes the rock body at the foot of the slope the most easily to be cracked and produce damage. Combining the previous studies with experimental phenomena, Fig. 9 and Fig. 19 shows that the foot of the slope is the first place where stress concentration occurs, and the first place to be damaged is the foot of the slope.

5. Lines 208-249: The authors describe the relationship between internal fracture characteristics and surface deformation, but most of the relationships remain at a qualitative level. Are there more quantitative analyses to determine the correlation between internal deformation and surface characteristics?

Answer：Dear reviewer, thank you for your question. In this paper, the qualitative analysis is carried out in the newly revised Chapter 4 (original lines 208-249) when describing the relationship between internal fracture characteristics and surface deformation, but in the newly revised Chapter 2, the relationship between internal fracture characteristics and surface deformation is quantitatively analysed in four aspects, namely, rate cloud map, cumulative displacement change characteristics, point displacement at each point of the 5 slab, and rate change at the point of the #1#, respectively, and at the same time, Fig. 12 demonstrates the internal and surface fracture development pattern of the slope.

6. Line 251: The section "Evolution trend of deformation indicators" is missing, please add it.

Answer: Thanks for your kind reminders. We have modified the order of the images by deleting section 3.4 and placing figures 7-10 from the section in the previous text. What was once Fig.7 is now Fig.5, what was once Fig.8 is now Fig.10, what was once Fig.9 is now Fig.6, and what was once Fig.10 is now Fig.12.

7. Lines 424-467: The validation of field data against model results should be elaborated. Explain how field observations support the model predictions and discuss any discrepancies.

Answer: Thanks for your kind reminders. We have made revisions and added relevant pictures of the site investigation and described them in the text to secondly improve the accuracy of the conclusions

8. Figure: Images should be self-explanatory, yet most images in this manuscript lack legends. I recommend the authors add legends to all images. Additionally, some images are unclear and excessive in number. It is suggested that the authors improve image clarity and consolidate similar images.

Answer: Thanks for your kind reminders, pictures have been modified

9. Line 25: Keywords serve to encapsulate the manuscript's content, enabling readers to swiftly identify and understand the topic and scope. In this manuscript, the current keywords do not adequately reflect the research subject. I suggest the authors revise them accordingly.

Answer: Thanks for your kind reminders. We changed the keywords to anti-dip stratiform rocky slope; centrifuge model test; early identification; macroscopic deformation characteristics

10. I do not understand the phrase 'which is situated in the best air condition.' Does this refer to the 'free face'?

Answer: Yes, we have changed my expression from 'the best air condition' to 'the free face'.

11. Lines 289-291: Regarding interlayer forces, many scholars have conducted research. In fact, the distribution of interlayer forces varies across different stages. Is it appropriate to assume a triangular linear distribution of interlayer forces in this context?

Answer: In this paper, it is proposed to "assume that the gravity loads act on the cantilever slabs in the form of triangular linear distribution of loads", and it is reasonable to assume the triangular linear distribution of interlayer forces for the following reasons: 1.Rock Layer Properties: The physical parameters (e.g., strength, deformability) of the rock layers in the centrifuge experiment do not vary significantly with depth. 2.Stress Distribution Theory: According to soil mechanics principles, the vertical stress distribution in a slope or foundation typically takes a triangular form. For an anti-dip slope, the interlayer force distribution, which represents the shear stress within the slope, can also be assumed to follow a triangular distribution. 3.Slope Geometry and Boundary Conditions: The geometry of the slope constructed in the centrifuge model is relatively regular, and the boundary conditions promote the interlayer force distribution to tend towards a linear triangular form, which is a reasonable assumption. 4.Centrifugal Loading: In the centrifuge model test, the primary load acting on the slope is the centrifugal force, which is relatively stable and does not exhibit pronounced dynamic effects. This allows the influence of the load to be simplified as a triangular linear distribution.5. Initial Deformation Stages: During the initial deformation stages (Stages I and II) of the slope, the deformation of the rock slabs is relatively small, and the interlayer force distribution remains close to the triangular linear assumption. This justifies the use of the "triangular linear distribution of gravity load on the cantilever slabs" to obtain the "ultimate displacement Umax" in these stages. 6.Slope Deformation Limit: As long as the cumulative deformation of the slope does not reach the limit value of deformation and instability (Umax), the slope will not progress into the more advanced stages of damage, even if it encounters various working conditions during its geological history. This further supports the reasonableness of the triangular linear distribution assumption for the interlayer force in the Stages I and II.

12. The literature review is required to be updated. Authors can use the following papers in the literature review.

(https://doi.org/10.1007/s12583-023-1835-1;https://doi.org/10.1016/j.enggeo.2023.107340;https://doi.org/10.1016/j.gsf.2022.101493 ; https://doi.org/10.1007/s11440-022-01627-0 ).

Answer: Sincerest thanks, which we have carefully read and cited. (LINE 47-51,68-74,59-66)

Reviewer #2:

1. This paper lacks a detailed introduction to actual landslides. In the centrifugal model test, the similarity ratio was 1:100. What is the size of the model box? What is the actual size of the landslide? Is there a review of the similarity ratio relationship?

Answer: Thanks for your kind reminders. We've added the size of the model box: length x width x height = 100 cm x 60 cm x 100 cm. The actual size of the landslide in the text: length 2949m, width 2496m.

2. The introduction section lacks some important references

(10.1007/s10064-021-02135-3;10.16285/j.rsm.2016.2518;10.1016/j.soildyn.2019.105961)

Answer: Thank you for recommending the literature, but this article is about loess landslides, so we reselected a 2024, a 2023 published literature on anti-dip stratiform rocky slope, read it carefully, and cited it. (LINE 81-87)

3. Many of the figures in the paper have inconsistent fonts, and some of them are not clear (Fig.2-8, 16, 20…).

Answer: Thanks for your kind reminders. We've rechecked all the images and revised it.

4. Some professional vocabulary is not used properly, for example, "front edge" is suggested to be changed to "toe".

Answer: Thanks for your kind reminders. We've changed my expression to make it more accurate and professional.

5. The author was suggested to supplement the sources, basic parameters, image time, and interpretation methods used for SAR images.

Answer: Thanks for pointing our question out, we've added the InSAR information to the article.

6. In addition to the deformation law, what is the relationship between the deformation amount and deformation rate of landslides obtained from model experiments and Insar data?
   Answer: Dear reviewers, I have the following answers to this question. The denser interferometric fringes and the larger colour difference in the Stacking-InSAR stacked phase map results mean that the deformation phase here is larger. According to the interpretation results of InSAR, in the process of Stacking-InSAR interferometric image identification, the effect of down-track identification is more obvious, the deformation is mostly concentrated in the steep and slow junction, and the deformation degree is greater near the top of the slope. The annual average deformation rate is greater. Since this paper is to study the relationship between the deformation pattern of the anticlinal laminated rocky slope and the macro deformation characteristics and did not carry out a detailed deformation rate interpretation of the InSAR data, only the deformation pattern of the Zayong deformed body was interpreted using the InSAR data to correspond with the deformation pattern of the modelling experiments, to confirm the feasibility of the study.
7. The author is suggested to add some textual descriptions in section 3.4.

Answer: Thanks for your kind reminders. We have modified the order of the images by deleting section 3.4 and placing figures 7-10 from the section in the previous text. What was once Fig.7 being now Fig.3, what was once Fig.8 being now Fig.10, what was once Fig.9 being now Fig.4, and what was once Fig.10 being now Fig.5.

8. The cumulative displacement thresholds of landslides lack detailed theoretical basis. Suggest supplementing relevant references.

Answer: Thanks for your kind reminders. We have added the corresponding references to the article, Paper. 23.

9. The conclusion is not concise and innovative. I believe that the Authors should try to interpret and explain more clearly their results. Some key quantitative conclusions should be supplemented.

Answer: Thank you for your suggestion, we have rewritten the conclusion to make it more concise highlighting its innovative nature.

10. In this study, the authors must improve the statements about what is new in their study and what are the contributions to the developments of landslide with toppling failure kinematic features.

Answer: Dear reviewer, thank you for your suggestion, we have further explained the new aspects of the research, contributing to the development of anticlinal rocky slopes, in the introductory part of the article as follows. Although previous researchers have carried out in-depth studies on the deformation evolution mode of dumping-fracture, there are still deficiencies in the corresponding relationship between the macroscopic deformation characteristics of the dumped deformed body and the internal rupture. In this paper, based on the previous research on monitoring and warning models, we carry out centrifugal model test research on anticlinal rocky slopes. We monitor the deformation of the deep rock slabs and the macro deformation characteristics of the ground surface during the test, divide the stages of rock dumping deformation, investigate the macro deformation characteristics of the ground surface corresponding to each stage, establish the corresponding coupling relationship between the dumping deformation pattern of the slope and the macro deformation characteristics of the surface, and guide the deformation results of the actual slope's InSAR interpretation and the actual field investigation, summarise the deformation characteristics of the slope and deformation rules, and visualize the deformation stages of the slope, to improve the deformation characteristics and internal rupture of the slope. The deformation stage of slopes can be visualised, thus improving the accuracy of early identification of such slopes and providing technical support for the identification, prevention and control of major landslide disasters.

Reviewer #3:

1. The title needs to be rewritten.

Answer: I've rewritten the title of the paper, changing it from ‘deformation stage division based on the surface behavior characteristics and internal rupture characteristics of toppling failure kinematic features’ to ‘Research on the relationship between the overturning deformation pattern and macro-deformation characteristics of anti-dip stratiform rocky slopes’.

2. When an author employs an abbreviation, the full term should appear at the first instance of the abbreviation.

Answer: Thanks for your kind reminders, we have revised it.

3. “tipping” is suggested to be “toppling”, please check the whole manuscript

Answer: Thanks for your kind reminders, we have revised it.

4. Insert a space between the unit and the number.

Answer: Thanks for your kind reminders, we have revised it.

5. Introduction- In fact, the research on the correlation between macroscopic deformation characteristics and internal fractures of anti-dip slopes has been quite comprehensive. On one hand, it is suggested that the authors include references from the past three years concerning studies on toppling failures. On the other hand, the innovative aspects of this paper need further enhancement to emphasize the originality and significance of this research.

Answer: Thanks for your kind reminders. We have added newer references on anticlinal rocky slopes in the last three years to the text, see line 47-51,68-74,59-66,81-87 in the text. In addition, I have rewritten the conclusion of this paper to make it more concise highlighting its innovative nature. This paper is innovative in the following ways: 1. Cumulative displacement is calculated for points with optimal critical conditions at the steep and slow intersection of landslides so that the critical threshold value of slope failure and its evolution can be used as an effective means of slope deformation stage delineation, and deformation monitoring. 2. This paper investigates the coupling between the deformation patterns of the inverted rock slopes and the macroscopic deformations of the surfaces and proposes the locations of key cracks on the surfaces, which can be used as an effective means for the division of the failure stages of slopes. 3. The InSAR interpretation and field investigations of the prototype slopes have also been used. 4. The InSAR interpretation and field investigation results of the prototype slope can be used to infer the slope stage, visualize the slope's deformation stage, and provide technical support for the identification, prevention, and control of this type of slope.

6. The manuscript has too many sections, please consolidate some sections to make the structure of the article more concise. For example, Sections 2.1 to 2.4 can be merged into one section.
   Answer: Thanks for your kind reminders, we have revised it.
7. Line 91 Wrong expression “Table 3Ⅰ-a” should be “Figure 3I-a”

Answer: Thanks for your kind reminders, we have revised it.

8. Line 98 “Observation points were equidistantly placed on the surface” Where are observation points located? Please mark the locations on the figure.

Answer: Due to the large number of observation points set up in this paper, plotting in Fig 2(a) is not aesthetically pleasing, and the detailed locations of the observation points are shown in Fig 14.

9. Section 2.3. Why the geometric similarity ratio is set of 1:100? What is the prototype rock type of the similar materials? Does the strength of the similar material meet the requirements of prototype rock? Please provide additional details in the manuscript.

Answer: We have added information in the text about the prototype slope, whose rock type is Chlorite Sericite Phyllite, and similar materials have been fabricated to match all physical parameters of the prototype rock

10. Line 130 “the max deviator stress（1−3）near the foot of the slope is the largest” . Please supplement the stress monitoring data to support this statement.

Answer: Dear reviewer, the pressure sensors of this experiment were all pulled out resulting in no valid pressure detection data collected. Based on this, this paper combines the Principles of Engineering Geological Analysis and Engineering Geology: Rock in Engineering Construction to obtain that in the process of slope formation, unloading rebound occurs in the rock mass around the critical surface, which causes stress redistribution as well as stress concentration effects. Under the influence of stress differentiation, a stress concentration zone is generated near the critical surface. The maximum principal stress near the foot of the slope increases and the minimum principal stress decreases, so that the difference between the maximum and minimum principal stresses here is the largest, which makes the rock body at the foot of the slope the most easily to be cracked and produce damage. Combining the previous studies with the experimental phenomena, Fig. 9 and Fig. 19 show that the foot of the slope is the first place where stress concentration occurs, and the first place to be damaged is the foot of the slope.

11. Lines 133-134 “While the small principal stress drops to zero or even changes to tensile stress, the maximum principal stress hits the highest point near the bottom of the slope.” Please supplement the stress monitoring data to support this statement.

Answer: Similarly, because the pressure sensors of this experiment were all pulled out resulting in no effective pressure detection data collected, this paper combines the Principles of Engineering Geological Analysis and Engineering Geology: Rock in Engineering Construction, the relationship between the maximum principal stress and the minimum principal stress is this and that, when the maximum principal stress is closer to the slope surface becomes larger while the minimum principal stress decreases to zero, gradually converting from compressive to tensile stress. the slope surface becomes larger while the minimum principal stress decreases to zero, gradually converting from compressive stress to tensile stress.

12. Line 146 Spelling mistake “slop” should be “slope”.

Answer: Thanks for your kind reminders, we have revised it.

13. Line 158. “Figure 8” should be “Fig. 7”. In addition, the expression of the figure should be standardized according to the journal's requirements.

Answer: Thanks for your kind reminders, we have revised it

14. Line 183 “No. 4” should be “No. 5”.
    Answer: Thanks for your kind reminders, we have revised it
15. Lines 234-235 “and tension cracks began to form at a degree of stress of about 1°.” I do not understand this sentence, especially the expression “degree of stress of about 1°”. More explanation needed to be added.

Answer: ‘degree of stress of about 1°’ means that the opening between the rock slab and the rock plate is about 1°. We've changed my expression from ‘Due to the centrifugal force, the rock slabs were fitted more closely, and tension cracks began to form at a degree of stress of about 1°’to ‘Due to the centrifugal force, the slabs fit more closely together and tensile cracks start to form when the tension between the rock slabs and the slabs is about 1°.’

16. The content in Section 3.4 is missing.

Answer: We have modified the order of the images by deleting section 3.4 and placing figures 7-10 from the section in the previous text. What was once Fig.7 is now Fig.3, what was once Fig.8 is now Fig.10, what was once Fig.9 is now Fig.4, and what was once Fig.10 is now Fig.5.

17. Line 309, “According to the previous centrifugal test, combined with Huang Runqiu’s deformation system”. Which research? What is Huang Runqiu’s deformation system? Please add related references and statement.

Answer: Thanks for your kind reminders. We've changed my expression from ‘previous’ to ‘above’ and added the reference to Paper.29. Table 1 shows the Huang Runqiu deformation system.

| Table 1 Evaluation system for slope dumping deformation | | | | | |
| --- | --- | --- | --- | --- | --- |
| Index | Extremely strong tipping overturn deformation A | | Strong dump rupture B | | Weak tipping transition deformation C |
|  |  |  | Upper section B1, cut layer shear rupture | Lower section B2, intra-laminar fissile deformation |  |
| Deformation rupture feature | The rock mass is strongly dumped, broken, and overturned, and the whole is relaxed, and the part is overhead | | The rock mass is strongly toppled, the layer is strongly stretched, the whole is relatively relaxed, and the tensile and shear fracture surface is developed | The dumping is relatively strong, the tensile rupture in the layer is stronger, and the tensile fracture surface is generally not cut into a layer, and a single layer is cut locally | The dumping deformation of the rock mass is weak, and it occurs between rock layers  The staggered deformation caused a slight tension phenomenon in the rock mass in the layer |
| Dip of rock formations  (Dump Angle)  α/° | Hard rock | ≤40（＞35~40） | 34~54（20~35） | 50~69（10~20） | 64~78（≤10） |
|  | Soft rock | ≤35（＞40） | 32~50（25~40） | 51~66（15~25） | 58~74（≤15） |
| Maximum tensor s/mm | Range value | 4.0~55.0 | 5.0~70.0 | 2.0~65.0 | 0~17.0 |
|  | average value | 28.0 | 26.0 | 15.0 | 5.0 |
| Unit tensor s/mm | Hard rock | 27.0~50.0 | 20.0~33.0 | 15.0~27.0 | 10.0~17.0 |
|  | Soft rock |  | 10.0~33.0 | 10.0~30.0 | 8.5~12.0 |
| Unloading deformation | Strong unloading | | Strong unloading | The overall strong unloading, the lower part can be weak unloading | Weak unloading |
| Weathering characteristics | Strong weathering | | Generally, the upper part is weakly weathered, and the upper part is strongly weathered | The upper part is weakly weathered, and the lower part is weakly weathered | The lower section of the overall weak weathering, some micro-new rock masses |
| Type of rock mass structure | Fragmentation of overhead structures, scattered structures | | Laminar mass-fractured structure | Laminar-block fissied structure | Laminar-like structure |
| Longitudinal wave velocity Vp/m∙s^-1^ | 1000~1500 | | 1500~2000 | 2000~3000 | 3000~4000 |
| Rock mass category | Ⅴ~Ⅵ | | Ⅲ_1_ | Ⅲ_2_ | Ⅱ |
| Analogue value of stability coefficient of rock mass category | 1.0~1.05 | | 1.05~1.15 | 1.15~1.25 (Separate evaluation if there is a broken surface) | ＞1.25 |

18. Line 318. “it is also necessary to subtract 5 cm” Why is 5 cm? It appears that the author intends to correlate the theoretical derivation results with the model test results. However, there seems to be a lack of scientific basis for this approach.

Answer: Thanks for your kind reminders, subtract 5 cm because point 1 is 5 cm from the surface.

19. Line 322 “Goodman and Bray proposed that the overturning deformation…”. Please add related references.

Answer: This is a duplicate of line 51, quoted in Paper 10, and we have modified the presentation to make it more succinct.

20. Line 345 The author did not label Zone B in Fig. 12.

Answer: This is a clerical error and we have changed ‘shown in Fig 12 above Zone B’ to ‘shown in Fig 14 above Zone B-T_B_’.

21. Fig. 17. What does the term” deformation intensity” mean? In addition, please add a legend to each contour map in Fig. 17.

Answer: Sorry, there is an error in my expression here. Figure 17 is the distribution of large deformation areas in each stage of landslide deformation based on the rate cloud characteristics and physical test phenomena, combined with the relative cumulative displacements of the rock slabs at different stages, which shows the transfer of large deformation areas of the anticlinal slope from the foot of the slope to the top of the slope stage by stage, reflecting the division of the main deformation areas in each stage of the landslide, as well as the relative degree of deformation of each area. I have added the corresponding legend to Figure 17.

22. Please add a scale bar to Fig. 21.

Answer: Dear reviewer, we have added a scale bar on the upper right corner of figure 21.

23. Line 457, The Zha Yong toppling deformation is in the III stage of slope dumping deformation but without explanation, please supplement. It is recommended that the authors supplement field photographs of slope deformation and compare them with physical model experiments to validate the accuracy of the conclusions. Currently, the classification of toppling evolution stages appears overly subjective, lacking quantitative criteria for assessment and support from field evidence.

Answer: We have added relevant images from the site investigation and described them in the text to secondly improve the accuracy of the conclusions.

24. Linking InSAR with the division of landslide evolutionary stages is a very interesting idea. However, in previous studies, the formation of large-scale toppling deformations in southwest China has been proven to occur during the valley evolution process. In another words, the toppling period is extremely long. Even if you obtain data for a period using InSAR, it is still very short relative to the existence time of toppling.

Answer: The long time series Stacking-InSAR deformation data can reveal the main deformation characteristics of landslides. Different stages of landslides have different deformation characteristics, such as in the creep stage, the slope shows small local displacements, and there is no obvious change in the geomorphological form; while in the sliding stage, the geomorphological form can already show the overall outline of the slope, and the slope disintegration phenomenon can be seen in the longitudinal direction, and the sliding speed of the landslide is gradually increasing.The monitoring data obtained by InSAR is indeed shorter than the period of tilting deformation of anticline slopes, but the anticline rocky slopes have ended their long-term creep phase under the long-term geological history, and Stacking-InSAR can observe the surface deformation in a wide range, retrospectively, and non-contactly, and it can obtain the centimetre or even millimetre-scale deformation of the ground, which can be widely applied to the monitoring of landslides. Stacking-InSAR is widely used in landslide monitoring. In the Stacking-InSAR stacked-phase map, the denser the interference fringes are and the larger the colour difference are, the larger the deformation phase is. Comparing the Stacking-InSAR stacked phase maps with Fig.5 and Fig.17 in this experiment corresponds to the third stage of deformation progression.

25. Language- in additon to the terminology issues, there are still a lot of problems regarding the English usage and the expression. The language at places is imprecise. The problems start from the first sentence of Abstract.

Answer: Dear reviewer, we have revised the article on the issues you raised and touched up the article.
