# Supplementary material for: Relationship between the surface and internal deformation features of toppling in anti-dip rock slopes
Source: PLoS One. 2024 Nov 7;19(11):e0312687. doi: 10.1371/journal.pone.0312687 (PMC11542880; doi:10.1371/journal.pone.0312687)
Supplement: Attachment — Submitted filename: Response to Reviewers.docx [file pone.0312687.s012.docx]

Response to Reviewers

**Additional Editor Comments:**

Reviewer #1:

1. The title might be better "Relationship between the overturning deformation pattern and macro-deformation characteristics of anti-dip stratiform rocky slopes" or "overturning deformation pattern and macro-deformation characteristics of anti-dip stratiform rocky slopes"

Answer：Thank you very much for your comments, and after my reflection, I changed the title of my paper from“Research on the relationship between the overturning deformation pattern and macro-deformation characteristics of anti-dip stratiform rocky slopes ”to“Relationship between the surface and internal deformation features of toppling in anti-dip rock slopes”

2. Summarized protocol for the physical model test is suggested (as shown in Table 10 at <https://doi.org/10.1016/j.gsf.2022.101493>)

Answer：Dear Reviewer,I have carefully studied the table 10 you provided for my paper, but I don't believe this paper is suitable for such a detailed description. The reasons are as follows: 1. This paper does not have that many parameters that need to be described. 2.The experiment only involved the application of gravity as a factor.3. Putting the experimental model parameters in a table format would not be able to intuitively demonstrate the construction of the experimental model.

Reviewer #3:

1. The title still needs to be revised. I recommend revising the title to:” Relationship between the surface and internal deformation features of toppling in anti-dip rock slopes”.

Answer：Thank you very much for your comments, and after my reflection, I changed the title of my paper from“Research on the relationship between the overturning deformation pattern and macro-deformation characteristics of anti-dip stratiform rocky slopes ”to“Relationship between the surface and internal deformation features of toppling in anti-dip rock slopes”

2. Please ensure consistency in the terminology throughout the entire text. For example:” dump” is suggested to be replaced by “topple”.

Answer：Sorry, I apologize for my mistake, I've replaced all the “dump”with “topple”and checked the full text.

3. I did not see any observation points in Fig. 14.

Answer：I apologize, but during the subsequent revision of the article, I changed the order of the figures, so the reference here should be to Figure 16, with the observation points being the black and white circular cross markers in that figure. As there are many observation points, directly depicting them in Figure 4(a) in the article would not be aesthetically pleasing. To facilitate your review, I have depicted them more clearly in Figure 16 and have also added a photograph of the actual model setup for comparison.

Fig.16

Actual piling photo

4. Fig.8d- 11d. Do the figures of “rate cloud diagram” refer to horizontal velocity, vertical velocity or total velocity? Is it the average velocity at that stage or instantaneous velocity? More details should be added.

Answer: The numbers in the rate cloud diagram refer to the average rate of total displacement change within a given stage. I have incorporated this clarification into the article text.

5. From Fig. 6, it is evident that the slope has already experienced failure when the acceleration reaches 48.9 g. However, as per design specifications, the stress field of the model can only match the actual conditions when the acceleration reaches 100 g. In such a scenario, can the results of the model tests truly represent the actual conditions?

Answer: Dear Reviewer, I have carefully considered the question you raised, and I will explain the reasons for the slope failure when the acceleration reaches 48.9g in the following text.

In actual rock masses, there are many cracks and joints, and the rock layer thickness is often not very thick, usually less than 50 cm. If the on-site rock layer thickness is scaled down at a ratio of 1:100, the experimental specimen would only be a few millimeters thick. During the model fabrication process, specimen boards with a thickness below 0.5 cm are very fragile and difficult to make successfully. In order to conduct the experiment, I increased the specimen thickness to 1 cm. In fact, even making 1 cm thick specimens was extremely challenging.

Moreover, the actual slope height is very high. Therefore, when constructing the model, we increased the thickness of the specimens. To correctly reflect the overall strength of the jointed and fractured rock mass, we appropriately reduced the strength of the individual specimens during the fabrication process.

The differential equations of flexural deformation for the rock plate in the model and the prototype are respectively:

$E_{m}I_{m}\frac{d^{4}x_{m}}{dy_{m}^{4}}+p_{m}=0$ （1.1）

$E_{p}I_{p}\frac{d^{4}x_{p}}{dy_{p}^{4}}+p_{p}=0$ （1.2）

*EI*represents the flexural rigidity of the rock plate; *E* represents the elastic modulus of the rock plate; *I* represents the moment of inertia of the cross-section of the rock plate. For a rectangular cross-section rock plate,$I=\frac{bh^{3}}{12}$, where *𝑏* is the width of the rectangular cross-section under bending, and *ℎ* is the thickness (height) of the rectangular cross-section in the bending direction.

According to the similarity theory (scaling law), the similarity ratios between the model (subscript *m*) and the prototype (subscript *p*) for the elastic modulus $E$, cross-sectional moment of inertia $I$, geometric dimension $l$, density $\rho$, and acceleration $a$ are respectivel

$S_{E}=\frac{E_{p}}{E_{m}}$，$S_{I}=\frac{I_{p}}{I_{m}}$，$S_{l}=\frac{l_{p}}{l_{m}}$，$S_{\rho}=\frac{\rho_{p}}{\rho_{m}}$，$S_{a}=\frac{a_{p}}{a_{m}}$

Substituting the above similarity ratios into Equation (1.2), it can be transformed as:

$S_{E}S_{I}E_{m}I_{m}\frac{s_{l}}{s_{l}^{4}}\frac{d^{4}x_{m}}{dy_{m}^{4}}+S_{\rho}S_{l}^{2}S_{\alpha}p_{m}=0$ （1.3）

Since Equation (1.2) and Equation (1.3) are both valid, the coefficients of the corresponding terms must be equal:

$\frac{S_{E}S_{I}}{S_{l}^{3}}=S_{\rho}S_{l}^{2}S_{\alpha}$ （1.4）

In the centrifuge model test, 𝑆𝜌=1，𝑆𝑎=1/𝑛，𝑆l=𝑛，therefore$S_{E}S_{I}=n^{4}$

That is, when the model and the prototype are similar, the flexural rigidity (EI) of the rock plate in the model and the prototype should satisfy the following relationship:

$E_{m}I_{m}=\frac{1}{n^{4}}E_{p}I_{p}$ （1.5）

In this experiment, the parameters of the model rock plate are *E_m_*=2800MPa, *b_m_* =10cm, *h_m_* =1cm. The prototype rock mass is moderately to slightly weathered phyllite, with an elastic modulus of *E_p_*=17.2GPa and geometric dimensions of *b_p_*=1000cm, *h_p_* =10cm.

Substituting these values into Equation (1.5), we get n≈49.8.

The bending and toppling mode not only needs to consider the geometric dimensions and the compressive and tensile strengths of the rock mass, but also the flexural rigidity. It is difficult to balance these three factors, as improving one aspect often compromises the other two. In this paper, the focus was on the geometric dimensions and the compressive and tensile strengths of the rock mass, while the flexural rigidity problem was overlooked. In fact, based on the above calculations, the flexural rigidity would fail at around 49.8g of centrifugal acceleration.

Considering the above factors, the concentrated deformation failure of the model occurred at around 50g.

Furthermore, the focus of our experiment is to reproduce the bending deformation process of the rock layers under anti-dip conditions, and to divide the deformation stages, so as to use the surface deformation characteristics as evidence to guide the InSAR interpretation. As long as the surface macroscopic deformation features and the rock layer bending deformation stages are consistent with the actual conditions, the results of this model test can be accepted and used.

6. Chapter 4, the logic is not sufficiently clear, and there is repetition among sections. The author can revise this chapter based on the following suggestions:

4.1 Evolution process of the toppling failure: Divide the toppling evolution stages based on the velocity field, deformation field, and the kinematic characteristics of rock columns, clearly outlining the criteria for stage division and the deformation characteristics of slopes in each stage;
4.2 Relationship between surface and internal deformation features: Focus on analyzing the surface and internal deformation characteristics at different evolution stages and their relationships.

Answer：Thank you very much for your suggestions. I have made the changes in the text accordingly.

7. Line 185 “Fig. 6Ⅱ-a”, maybe “ Fig. 11Ⅱ-a”

Answer：Thank you for pointing out my mistake. I have changed the label from “Fig. 6Ⅱ-a” to “ Fig. 11Ⅱ-a”and apologize for my oversight.

8. I recommend that the authors provide some practical case studies in the study area to validate the accuracy and practicality of the proposed methods in section 5.2.

Answer：Thank you very much for your suggestions. Regarding the verification of the method proposed in Section 5.2, this paper compares the results calculated based on $U_{MAX}$ with the maximum value of the cumulative displacement curve (Figure 7) of point 1 at the time of slope failure, and finds that the two results are similar. This verifies the reasonableness of the $U_{MAX}$ inference, thereby confirming the accuracy and practicality of the method proposed in Section 5.2.

9. What does the “distance” in Fig. 17 represent?

Answer：The distance here represents the distance between the selected point T and the toe point B. For example, when the distance is indicated as 20, it represents the distance between the yellow point and the green point at the slope toe.

Distance Schematic Diagram

10. I did not see any legends in Fig. 19

Answer：I'm very sorry, in the previous version I uploaded an old version of the figure. I have now updated figure 19

Fig. 19

11. It is advisable to consolidate Chapters 4 and 6.

Answer：Dear Reviewer,Thank you for your valuable suggestions. Chapter 4 focuses on describing the correspondence between the macro-deformation characteristics of the surface layer, the experimental phenomena, and the image analysis of the centrifuge model test data. Chapter 6 summarizes the correspondence between the toppling deformation patterns and the macro-deformation characteristics of the anti-dip rock slopes. Chapter 6 is derived from Chapter 4. Many of the sliding rules of anti-dip rock slopes are summarized from the centrifuge model tests, moving from specific to general. Based on the centrifuge model tests, the development process of key surface cracks is proposed as a monitoring means for the deformation and failure of such slopes. Combining the characteristics of the velocity contour maps, the physical test phenomena, and the relative cumulative displacement of rock plates at different locations, the deformation schematic diagrams for each stage of the toppling deformation are obtained. In summary, I'm afraid I cannot merge Chapters 4 and 6, but I have reduced the content of Chapter 6 to make the expression more concise.

12. I recommend changing the title of Chapter 7 to "Case Study”. In this chapter, the authors can validate the correspondence between surface and subsurface deformation characteristics from the previous test and the accuracy of the proposed evaluation methods in chapter 5.

Answer：Thank you for your suggestions. However, the primary purpose of this paper is to establish the coupling relationship between the slope deformation patterns and the surface macroscopic deformation characteristics. By integrating InSAR remote sensing interpretation and actual ground investigation, we can infer the stage of the slope and provide technical support for the early identification and prevention of major landslide disasters. The landslide case study in Chapter 7 is the specific example used for the centrifuge model experiments in this paper. The purpose of writing Chapter 7 is to verify that the research on the toppling deformation patterns of anti-dip rock slopes and their corresponding surface macroscopic deformation characteristics can guide the InSAR remote sensing interpretation. Due to the above reasons, I have not made any changes to the title of Chapter 7.

13. Language- Partly improved. But there are still some problems regarding the English usage. Below are just few examples. However, the entire text, including figure captions, has to be carefully edited.
    (1) Lines 111 “ deformers” bad expression.
    (2) Lines 112 ”slope body” bad expression.

Answer：Thank you for your suggestions. I have re-polished the language in the article.
