# Supplementary material for: Relationship between the surface and internal deformation features of toppling in anti-dip rock slopes
Source: PLoS One. 2024 Nov 7;19(11):e0312687. doi: 10.1371/journal.pone.0312687 (PMC11542880; doi:10.1371/journal.pone.0312687)
Supplement: Attachment — Submitted filename: Response to Reviewers.docx [file pone.0312687.s013.docx]

Response to Reviewers

**Additional Editor Comments:**

Reviewer #3:

1. The legend for the deformation intensity in Figure 19 should be quantified, and there is a lack of units to represent the deformation intensity. In addition, the concept of deformation strength intensity should be explained in the text.

Answer：Figure 19 is based on the characteristics of the velocity cloud and the physical test phenomena, combined with the relative cumulative displacement of the rock plates at different stages. It shows the gradual transfer of the main deformation zone from the slope toe to the slope top on the anti-dip stratiform rocky slopes. In fact, Figure 19 is a generalization of the velocity cloud diagram, representing the changing trend of the main deformation zone distribution at different stages of this type of toppling deformation body, as well as the relative deformation severity in each region. The reason why the legend cannot be quantified in this paper is that Figure 19 represents a type of landslide rather than a specific one, so the different colors in the legend only represent the varying degrees of deformation severity, from red to blue indicating a decrease from severe to mild deformation. To better represent the changing trend of the deformation zone distribution, I have changed the title of Figure 19 to "Distribution of the severity of toppling deformation".

2. The expression of “distance” in Fig. 17 should be added in the text.

Answer：Dear Reviewer, thank you for the reminder. I have added the explanation of the meaning of "Distance" in Figure 17 into the main text.

3. The language of the manuscript still needs further enhancement

Answer：Dear Reviewer, I have carefully re-polished the entire manuscript. I hope it can meet your approval.
